# The Potential Global Distribution of *Sirex juvencus* (Hymenoptera: Siricidae) under Near Current and Future Climatic Conditions as Predicted by the Maximum Entropy Model

**DOI:** 10.3390/insects12030222

**Published:** 2021-03-05

**Authors:** Tai Gao, Juan Shi

**Affiliations:** Sino-France Joint Laboratory for Invasive Forest Pests in Eurasia, Beijing Forestry University, Beijing 100083, China; gaotai36@outlook.com

**Keywords:** *Sirex* wood wasp, MaxEnt, potentially suitable area, climate change, CMIP6, co-infestation, pest control

## Abstract

**Simple Summary:**

The difference in the potential distribution of *Sirex juvencus* (Hymenoptera: Siricidae) under current and future climatic conditions is dependent on environmental factors such as temperature and precipitation, which can affect survival. In this study, we investigated the impact of climate change on the distribution and spread of an invasive insect pest species of forestry, *S. juvencus*. This wood wasp drills holes on a tree trunk or branch with ovipositor and then deposits eggs inside the hole, along with a wood-rotting fungus which helps its larva to digest the host plant. We analyzed the current distribution data from Asia, Europe, and North America with the maximum entropy model and revealed that the species might increase its distribution area as ambient temperature increases and precipitation (moisture) declines. There is also evidence to show that the species will spread more in moderately suitable areas. The species can also co-infest hosts along with other *Sirex* species of wood wasp, making its potential impact highly significant.

**Abstract:**

Wood wasp species in the genus *Sirex* are known pests of forestry. They cause significant economic losses due to their impacts on plant health and wood quality. *S. juvencus* (Hymenoptera: Siricidae), widely distributed in Asia, Europe, and North America, is known to negatively impact forestry, infesting *Picea*, *Pinus*, *Larix*, *Abies*, *Cupressus*, and *Pseudotsuga* species. This pest destroys plants by depositing eggs, mucus, and its obligate mutualistic fungus, *Amylostereum areolatum.* Its obligate mutualistic fungus is to provide nutrition for *S. juvencus* larva. Despite its extensive distribution range, little is known about which environmental variables significantly impact current and future distribution patterns of *S. juvencus* for pest control and monitoring. Here we used the maximum entropy model in conjunction with occurrence points of *S. juvencus* and environmental variables to predict the current and future global potential distribution of *S. juvencus*. We used the jackknife method and Pearson’s correlation analysis to select the environmental variables that influence the geographic distribution of *S. juvencus*, which resulted in the inclusion of the monthly average maximum temperature in February, the max temperature of warmest month, monthly average minimum temperature in July, monthly total precipitation in June, precipitation of the driest month, monthly total precipitation in September, and the temperature annual range. Temperature and precipitation are mainly likely to drive the distribution enabled by its obligate mutualistic fungus and the potential to co-infect with other *Sirex* species. The high temperature and low humidity influence *S. juvencus* eggs and larvae directly and indirectly via fungus-growth, which enables the larvae to survive. Furthermore, *S. juvencus* may increase its distribution to moderately suitable areas due to competition or dependency on other *Sirex* species during the infestation. Under the future climatic conditions, the highly suitable area increased by 32.79%, while the moderately suitable area, low suitable area, and unsuitable area increased by 28.14%, 3.30%, and 2.15%. Under climate changes, *S. juvencus* may spread in previously unsuitable areas rapidly.

## 1. Introduction

*Sirex juvencus* (Hymenoptera: Siricidae) is a widely distributed wood-boring wasp species in Canada, the U.S., Mongolia, China, Japan, Europe, and some west Asian countries [1,2,3,4,5,6,7,8,9,10,11,12,13,14,15,16,17,18,19]. As with other *Sirex* species, it is an important pest of conifers with high ecological and commercial value [20]. The *Sirex* species’ life cycle is about one year, but *S. juvencus* can be up to two years. For males and females, the emergence period of *Sirex* species tends to be close, even though the males emerge earlier. By using the ovipositor, the female pierces the host plant, drilling through the phloem to the xylem, with multiple tunnels in the xylem during oviposition. During oviposition, the female *S. juvencus* not only deposits eggs into the tunnel, but also injects the obligate mutualistic fungus, *Amylostereum areolatum* [21], and the mucus into other tunnels. The mucus contains a phytotoxin that promotes the growth of the fungus. Its obligate mutualistic fungus can weaken the host plant’s immune system and destroy the cellulose and lignin [22]. Researchers have shown that neither mucus nor its obligate mutualistic fungus can directly cause tree death alone. It is only the combination of mucus and its obligate mutualistic fungus that can cause the tree to be weakened or die [23,24]. The mucus and its obligate mutualistic fungus have severe negative impacts on wood quality and ultimately reduce the wood’s commercial value. Once a tree is infested, it can never recover again; this is a substantial economic loss [25,26].

Compared to other *Sirex* species, *S. juvencus* has a longer lifecycle and shows a preference for weakened or dying trees that other *Sirex* species have infested. Still, it can also infest living spruce [26]. This is a unique feature of this particular species. Furthermore, its distribution is known to be in Asia, Europe, and North America, but its potential to spread to other regions has not been investigated before. Considering the impacts of climate change and prevalence of *S. juvencus*, it is essential to ascertain what effects will result from changes in environmental factors and how these could influence the spread of invasive alien species (IAS), as well as to assist in the development of a monitoring and evaluation strategy [27,28].

Under global climate change, previously unsuitable habitats may become more habitable [29]. The model used in most of these studies uses data on climatic variables in the current occurrence areas and some of the species’ characteristics to predict possible suitable habitats under various climate scenarios. Maximum entropy theory is one such model and predicts species’ potential distribution area using occurrence points and environmental variables [30,31,32,33]. And the maximum entropy model has been applied in various studies predicting habitat suitability for plants, animals, and fungi [34,35,36,37], especially in invasion biology studies. The maximum entropy model is highly advantageous above other models due to its faster operational capability, simplicity of operation, stable calculation results, and high accuracy [38]. The maximum entropy model is ideal for studying the distribution patterns of *Sirex* wood wasps since they occur in areas that are poorly accessible, making it challenging to collect occurrence data due to their extensive habitat ranges.

This study aimed to predict the potential global geographic distribution of *S. juvencus* and the environmental variables driving this under near current and future climate scenarios using the maximum entropy model and the Coupled Model Intercomparison Project Phase 6 (CMIP6) data. The environmental variables used in this study are download from the WorldClim v2.1 (https://www.worldclim.org/) (accessed on 4 March 2021). We used the environmental variables of the future climate condition, the periods of the 21st century, under four shared socio-economic pathways (SSPs), including ssp126, ssp245, ssp370, and ssp585. We chose the medium-resolution National (Beijing) Climate Center Climate System Model (BCC–CSM2–MR), as the global climate model (GCM), to predict the potential distribution areas of *S. juvencus* under future climate conditions. We attempt to understand the factors that could drive the species to prefer weakened or dying trees to infest and explain its current global distribution relative to other *Sirex* species.

## 2. Materials and Methods

### 2.1. The Source of Occurrence Points

The occurrence data of *S. juvencus* were obtained from the following sources: (1) the collection records of *S. juvencus* specimens deposited in Beijing Key Laboratory for Forest Pests Control (Beijing Forestry University, BFU, Beijing, China), Unité de Recherche de Zoologie Forestière d’Orléans (l’Institut National de Recherche pour l’Agriculture, l’Alimentation et l’Environnement, INRAE, Orléans, France), National Zoological Museum of China (Chinese Academy of Science, CAS, Beijing, China), and Research Institute of Forest Ecology, Environment, and Protection (Chinese Academy of Forestry, CAF, Beijing, China); (2) published references related to *S. juvencus* [1,2,3,4,5,6,7,8,9,10,11,12,13,14,15,16,17,18,19]; (3) the pest’s distribution databases including GBIF (https://www.gbif.org/) (accessed on 4 March 2021), CABI (https://www.cabi.org/) (accessed on 4 March 2021), INPN (https://inpn.mnhn.fr/accueil/index) (accessed on 4 March 2021), NBN Atlas (https://nbnatlas.org/) (accessed on 4 March 2021), and PESI (http://www.eu-nomen.eu/portal/index.php) (accessed on 4 March 2021); (4) the observational data collected from 2016 to 2020, with which we investigated 83 historical and potential occurrence points in China and Europe. Finally, we have collected 651 occurrence points of *S. juvencus* from the above sources, distributed in Asia, Europe, and North America.

### 2.2. The Selection of Occurrence Points

We used Google Earth 7.1 (Google Inc., Mountain View, CA, USA) to check the latitude and longitude of the collected occurrence points. To eliminate the influence of large spatial correlation and over-fitting simulation, we used the buffer analysis to filter the occurrence points that were too close. The spatial resolution of environmental variables used in this study was 2.5 arc-minutes, covering about 21 km^2^, with the buffer zone’s radius set to 2.5 km. Only one occurrence point was retained when the distance between the occurrence points was less than 5.0 km [39].

### 2.3. Environmental Variables

Insect species’ distribution patterns are influenced by various environmental variables [40]. The environmental variables used in this study can be seen in Table 1. We used the ArcMap software v10.2 (Environmental Systems Research Institute Inc., Redlands, CA, USA) to convert the environment variables from *.tif format to *.asc format, in preparation for use in the MaxEnt software 3.4.1 (Princeton University, Princeton, NJ, USA). The coordinate system was set to WGS 1984. Since too many variables can increase the ecological space dimension, which can then affect the predicted performance and accuracy of the maximum entropy model, we used the jackknife analysis in the MaxEnt software to obtain the percent contribution of each environmental variable [41,42,43]. We extracted the environmental variables’ climate information of all occurrence points using the ArcMap software. We used Pearson’s correlation analysis to calculate the relationship between the climatic and environmental variables using the SPSS software v20.0.0 (International Business Machines Corporation, Armonk, NY, USA) [44].

### 2.4. Maximum Entropy Model Optimization

The maximum entropy model parameters set in this study were as follows: create response curves and jackknife to measure variable importance; cloglog output mode [33]; output file format as *.asc format. For predicting the potentially suitable distribution area under future climate conditions, the filenames of future climate variable data were set to correspond to the near current climate variables and loaded into “Projection layers directory/file”.

The following parameters were also used in the model analysis. We used random seed, which is similar to bootstrapping, where each run will use a different random seed. The random test percentage was set to 25, which means that 75% of the occurrence points are randomly selected as the training set, and the remaining 25% are used as the test set. We used the ArcMap software, DIVA-GIS software v7.5.0 (https://www.diva-gis.org/) (accessed on 4 March 2021), and R software v4.0.3 (https://www.r-project.org/) (accessed on 4 March 2021) to calculate the regularization multiplier (RM). The R packages ENMeval, dismo, dotCall64, fields, grid, knitr, maps, maptools, raster, rgeos, sp, spam, and spThin were used to calculate the RM and feature classes (FC). The RM was set to 0.5, 1.0, 1.5, 2.0, 2.5, 3.0, 3.5, and 4.0. The FC included linear (L), quadratic (Q), hinge (H), product (P), and threshold (T). The ENMeval package was used to calculate the corrected Akaike information criterion correction value (AICc value) under different parameters. The AICc value was used to estimate the maximum entropy model’s complexity, with the smallest AICc value corresponding to the FC combination and preferred RM [45]. We tested eight different FC combinations, including L, LQ, LQP, QHP, LQH, LQHP, QHPT, and LQHPT [32,46,47]. The checkerboard2 method was used to calculate the AICc value. The model was run 20 times under the same setting to further reduce the randomness of predicted results, with the final result presented as the replicates’ average [48].

### 2.5. Classification of Potentially Suitable Areas

The *.asc format file resulting from 20× replication of the maximum entropy model was converted to a raster by ArcMap. The worldwide distribution range of *S. juvencus* was analyzed using ArcMap. This study used the lowest presence threshold (LPT) to define the suitable distribution area and unsuitable distribution area [49]. The contents of the potential distribution areas were divided into four categories including unsuitable areas (0–LPT), low suitable areas (LPT–0.4), moderately suitable areas (0.4–0.6), and highly suitable areas (0.6–1.0).

### 2.6. The Predictive Accuracy of the Maximum Entropy Model

The area under the receiver operating characteristic (ROC) curve (AUC), the AUC value, is widely used to estimate the predictive accuracy of the maximum entropy model [50,51,52]. The following criteria are used to determine if the predicted accuracy has no predictive ability or a high predictive ability: AUC value range from 0.5 ≤ to ≤ 1; the output showing 0.5 ≤ AUC ≤ 0.6 is considered no predictive ability; 0.6 < AUC ≤ 0.7 is a poor predictive ability; 0.7 < AUC ≤ 0.8 is a general predictive ability; 0.8 < AUC ≤ 0.9 is a moderate predictive ability; 0.9 < AUC ≤ 1.0 is a high predictive ability. To eliminate the possible deviations of the AUC value, we used the AUC value of the partial-area ROC (P-ROC AUC) to estimate the predictive accuracy of the maximum entropy model. We used a 5% error rate (E = 0.05) to calculate the AUC ratios, AUC ratio = AUC_E_/AUC_0.5_, by using the Niche Analyst software v3.0 (http://nichea.sourceforge.net/) (Calculate AUC value of partial—area ROC approaches). The AUC ratio > 1 indicated that the model has a high credibility [53]. Moreover, we used a minimum training presence omission rate (ORmtp), 0 as expected value, and 10 percentile training presence omission rates (OR10), 0.1 as the expected value, to verify whether the model was overfitting [30,49,54]. When the model has a higher AUC value and a lower omission rate, it means it has a high predictive ability.

## 3. Results

### 3.1. The Major Parameters of the Maximum Entropy Model

**Figure 1:**
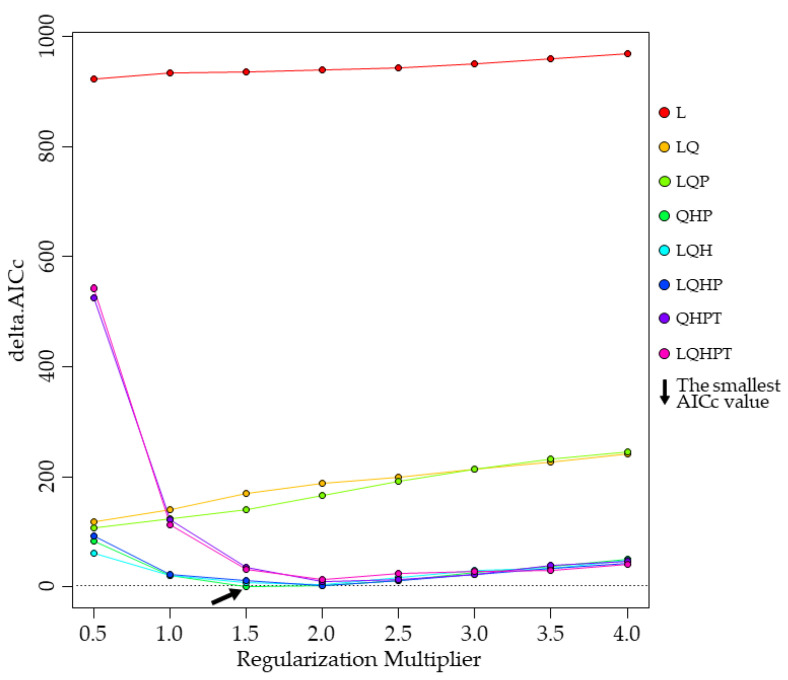
The regularization multiplier and feature classes of *S. juvencus* in the maximum entropy model.

We used buffer analysis to all collected 651 occurrence points and finally got 270 occurrence points of the *S. juvencus*, distributed in Asia, Europe, and North America, for the maximum entropy model. By using the jackknife analysis and Pearson’s correlation analysis to select bioclimatic/environmental variables, the results of selected environmental variables are shown in Table 2. The FC combination of the maximum entropy model in this study was QHP and the RM was 1.5 (Figure 1).

### 3.2. The Predictive Accuracy of the Maximum Entropy Model

**Figure 2:**
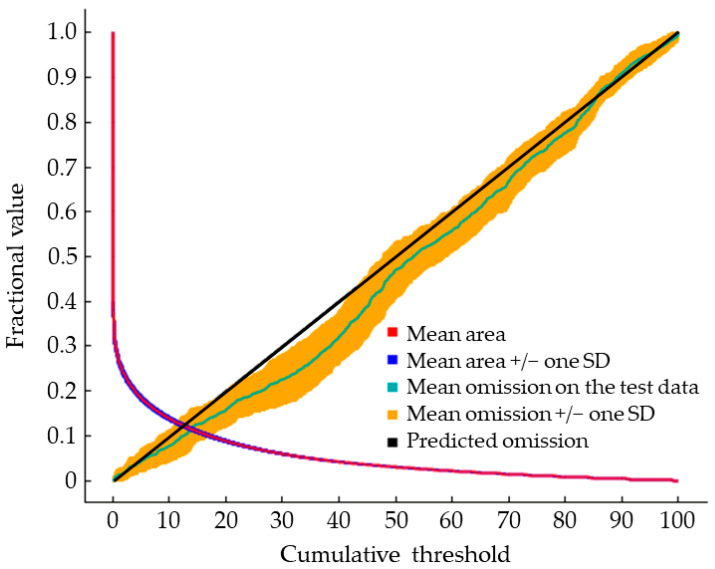
The output of the maximum entropy model’s predictive accuracy under near the current climate condition.

Based on the data of 270 occurrence points of *S. juvencus* and environmental variables, we used the maximum entropy model to predict the worldwide potential distribution area of *S. juvencus*. The accuracy of the predicted results was estimated using the AUC value, ORmtp, OR10, and AUC ratio under near current climate and future climate extrapolations. The results showed that the AUC and AUC ratio values of 17 climate conditions were all greater than 0.9 and 1.0, respectively. And the ORmtp and OR10 were all close to their expected value, 0 and 0.1, respectively (Table 3). Under near current climate conditions, the mean omission curve on the test data only had a very slight deviation near the predicted omission (Figure 2). The above results indicated that the maximum entropy model’s predicted results had a high predictive ability.

### 3.3. The Potential Distribution of S. juvencus under the Near Current Climate Condition

**Figure 3:**
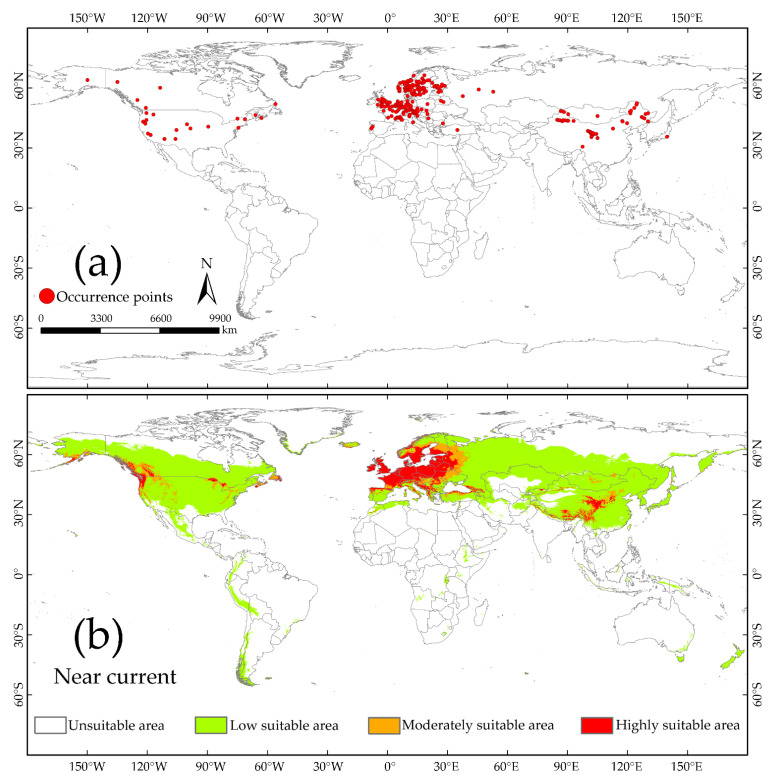
(**a**) Current occurrence and global distribution of *S. juvencus*; (**b**) The predicted area suitability under the near current climate condition. *S. juvencus* has the potential to occupy over 33.55% of the land area of the world except for the unsuitable continent, Antarctica, which shows high suitability for the occurrence of *S. juvencus*.

The 270 occurrence points of *S. juvencus* were distributed in the Northern Hemisphere: 8° W–53° E, 39°–66° N in West Eurasia; 85°–140° E, 31°–52° N in East Asia; 56°–150° W, 35°–64° N in North America (Figure 3a). The potential global distribution of *S. juvencus* under near current and future climatic conditions generated base on minimum training presence Cloglog threshold. The LPT for *S. juvencus* is 0.0165. The potentially suitable distribution areas of *S. juvencus* as predicted by the maximum entropy model were divided by LPT into four grades: highly suitable, moderately suitable, low suitable, and unsuitable (Figure 3b). The predicted results showed that the suitable area range is mainly distributed in the Northern Hemisphere under the near current climate condition. The highly suitable area in East and Central Asia is 70°–161° E, 26°–55° N; in Europe, West Asia, and North Africa it is 22° W–48° E, 31°–71° N; in North America it is 53°–164° W, 31°–62° N. Areas predicted to be highly suitable areas in Asia included Japan, Russia, China, Mongolia, Myanmar, India, Bhutan, Nepal, Pakistan, Afghanistan, and Kyrgyzstan. With the exception of Moldova, the potentially suitable area of *S. juvencus* was distributed in all countries in Europe. In Central Asia, *S. juvencus* was distributed in Armenia, Azerbaijan, Georgia, Turkey, and Iran, while in North Africa, it was distributed in Algeria and Morocco. In North America, *S. juvencus* was distributed in Canada, the U.S., and Mexico. The moderately suitable areas were mainly distributed around or near highly suitable areas (Figure 3b). In Eurasia, North America, western South America, and eastern Oceania were distributed great-area continuous low suitable areas. In contrast, in some regions, including east-central Africa, western South America, Southern Asia, and northern Oceania, the low suitable areas were sporadically distributed (Figure 3b). Of the total 270 recorded occurrence points, 70.74% (191) were assigned to highly suitable areas, followed by 19.26% (52) to low suitable areas, and 10.00% (27) were assigned to moderately suitable areas (Figure 3). No occurrence points were distributed in the unsuitable areas predicted under near current climate conditions.

**Figure 4:**
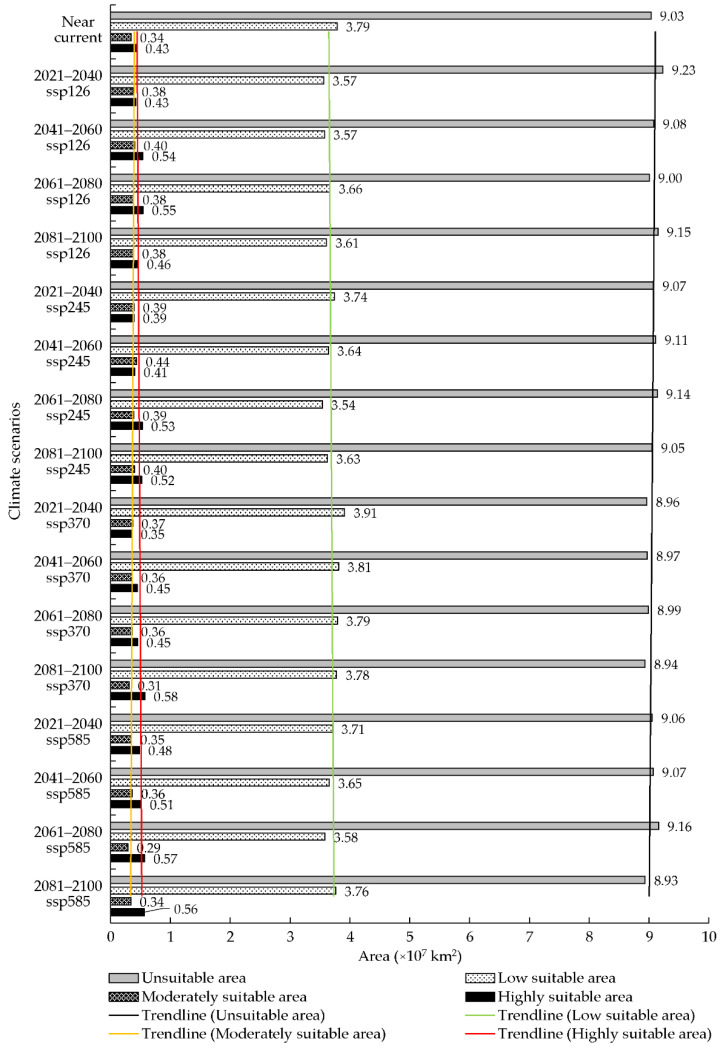
The worldwide suitable areas of *S. juvencus* under the near current and future climate conditions.

Globally, there is a potential for the spread of *S. juvencus*. The predicted highly suitable area for *S. juvencus* was 0.43 × 10^7^ km^2^, accounting for 3.16% of the total land area; followed by the moderately suitable area covering 0.34 × 10^7^ km^2^ and accounting for 2.50% of the total land area under near current climate conditions (Figure 3b and Figure 4). A large part of the world remains unsuitable for *S. juvencus* (Figure 3b and Figure 4). Highly suitable areas, and moderately suitable areas, increase by between 0.35 and 0.58 × 10^7^ km^2^, 0.29 and 0.44 × 10^7^ km^2^, respectively, under future climate scenarios (Figure 4). The low suitable areas do not show any significant increase under various future climate conditions (Figure 4).

### 3.4. The Relationship between the Distribution of S. juvencus and the Environmental Variables

**Figure 5:**
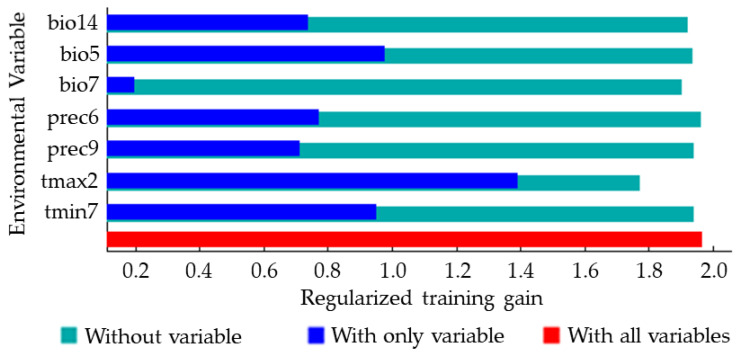
Jackknife analysis result showing the most influential environmental variables predicting potentially suitable distribution areas of *S. juvencus* around the world. The monthly average maximum temperature in February (tmax2) significantly contributed to explaining the suitability of the area for *S. juvencus*. And tmax2 has the most information that is not present in the other environmental variables.

The jackknife method was used to analyze the importance of the seven environmental variables that strongly impacted the distribution of *S. juvencus* (Figure 5). The longer the blue bar is, the more influential the variable is to the species distribution, and the shorter the green bar is, the more information the variable has compared to others (Figure 5). Among the seven environmental variables, the two environmental variables that have a greater impact on the distribution of *S. juvencus* were monthly average maximum temperature in February (tmax2), max temperature of the warmest month (bio5), and monthly average minimum temperature in July (tmin7) (Figure 5). Besides, the influence of the bio5 and tmin7 are very close. The monthly average maximum temperature in February (tmax2) provided the most information with regard to the distribution of *S. juvencus*, contributed significantly, and gave unique information in predicting area suitability.

**Figure 6:**
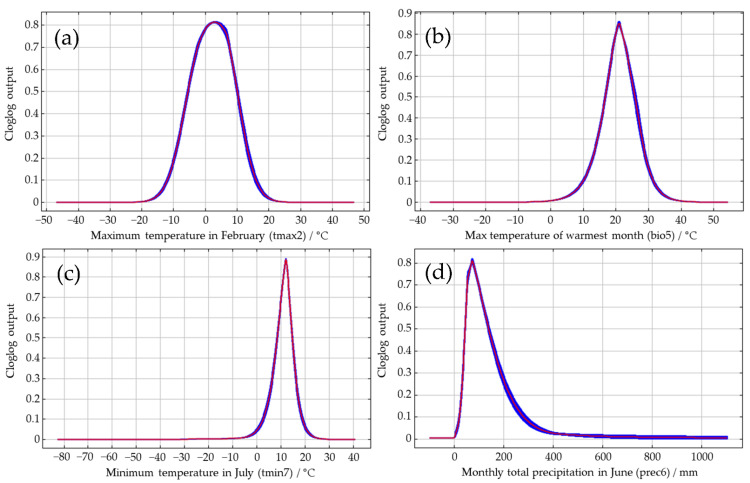
Response curves between the distribution probability of *S. juvencus* and environmental variables: (**a**) Maximum temperature in February; (**b**) Max temperature of warmest month; (**c**) Minimum temperature in July; (**d**) Monthly total precipitation in June. Values shown are average over 20 replicate runs: blue margins show ± SD calculated over 20 replicates.

The response curves between the dominant environmental variables and the distribution probability drawn by the maximum entropy model (Figure 6) reflected the range of environmental variables under different thresholds. In this study, the lowest presence threshold (LPT) was used to divide the range of potentially suitable distribution areas of *S. juvencus*. The results showed that the suitable temperature under tmax2 ranged between −16–20 °C, and the most suitable value was 3 °C. The suitable temperatures in the highly suitable areas ranged between −4–8 °C. The probability of occurrence increased with increasing temperatures when tma2 was between −16–3 °C, and decreased with increasing temperatures when tmax2 was between 3–20 °C (Figure 6a). The suitable temperature range for the highly suitable area was predicted to be 17–24 °C with a suitable bio5 value of 3–36 °C, and the most suitable value was found to be 20 °C (Figure 6b). When bio5 was between 3–20 °C, the probability of occurrence increased with increasing temperatures, and when bio5 was between 20–36 °C, the probability of occurrence decreased with increasing temperatures. The suitable value for the highly suitable area was predicted to be between 9–14 °C with a suitable tmin7 value of −3–23 °C, and the most suitable value was found to be 12 °C (Figure 6c). The probability of occurrence increases as temperature increases when tmin7 was between −3–12 °C and decreases with increasing temperatures when tmin7 was between 12–23 °C. The suitable value of prec6 for the highly suitable area was between 49–117 mm, with a prec6 of between 4–520 mm (Figure 6d). The probability of occurrence increases with increasing precipitation when prec6 ranged between 4–73 mm, and decreases as the precipitation increases when the precipitation was between 73–520 mm. Thus, areas of excess temperature or precipitation could negatively influence the establishment of *S. juvencus*.

### 3.5. Potential Distribution of S. juvencus under Future Climate Conditions

**Figure 7:**
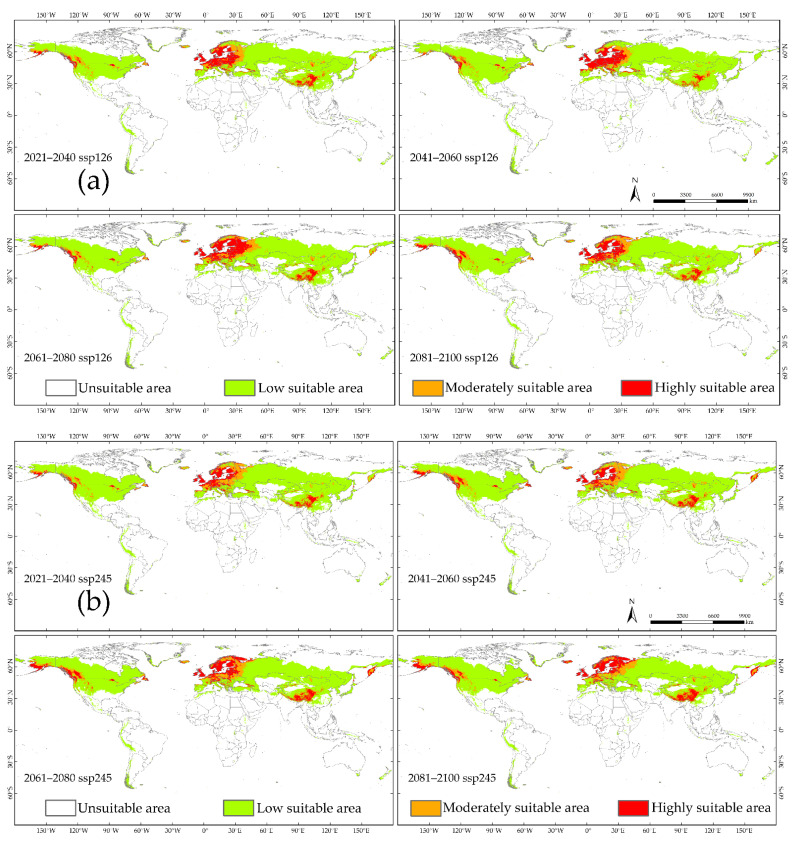

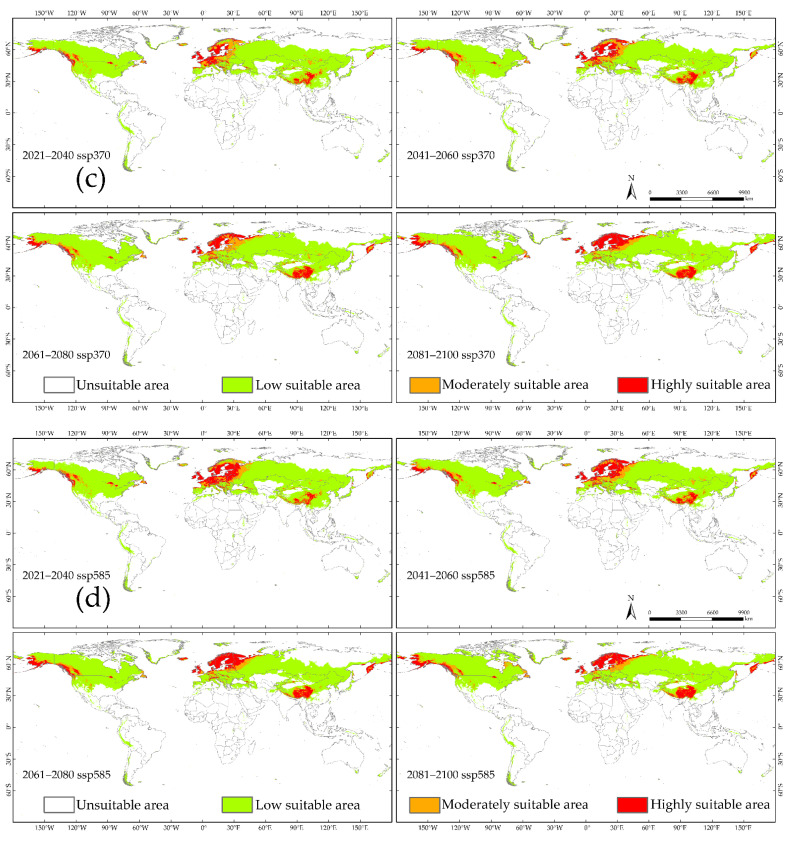
Predicted potential distribution of *S. juvencus* around the world under the future climate conditions during the periods of the 21st century under four CMIP6 climate scenarios and four shared socio-economic pathways: (**a**) ssp126; (**b**) ssp245; (**c**) ssp370; (**d**) ssp585.

**Figure 8:**
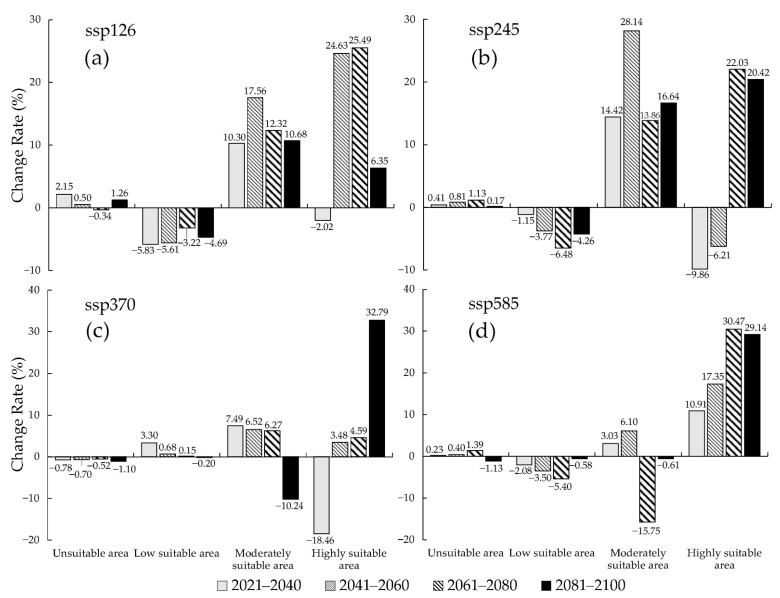
The change rate of predicted potential distribution of *S. juvencus* around the world under the future climate conditions during the periods of the 21st century under four CMIP6 climate scenarios, compared with the potential distribution area under near current climate conditions and four shared socio-economic pathways: (**a**) ssp126; (**b**) ssp245; (**c**) ssp370; (**d**) ssp585.

Under the future climate conditions, the periods of the 21st century 2021–2040, 2041–2060, 2061–2080, and 2081–2010, under four CMIP6 climate scenarios including ssp126, ssp245, ssp370, and ssp585, showed an increase in predicted area suitability for the distribution of *S. juvencus* around the world. There is a high potential for *S. juvencus* to spread into unsuitable areas which are becoming suitable under climate change (Figure 4, Figure 7 and Figure 8). There is an increasing spread of highly suitable areas for the occurrence of *S. juvencus* globally, especially in the northern parts of the world (Figure 7a–d). Moderately suitable and highly suitable areas show an increasing trend under the ssp126 of the future climate conditions in the periods 2061–2080 and 2081–2100 (Figure 7a), during the periods 2041–2060 and 2081–2100 under the ssp245 (Figure 7b), during the periods 2061–2080 and 2081–2100 under the ssp370 (Figure 7c), and during the periods 2041–2060, 2061–2080, and 2081–2100 under the ssp585 (Figure 7d).

The increasing trend of highly suitable areas is evident under various future climate scenarios (Figure 8). Moderately suitable areas show an increasing trend under future climate scenarios 2081–2100 ssp126 and ssp245 (Figure 8a,b). The maximum observed increase in the predicted highly suitable area appears in the period 2081–2100 ssp370, reaching an area of 0.58 × 10^7^ km^2^, with an increase of 32.79% compared to that under near current climate conditions (Figure 8c) and an increasing trend for the same period under ssp245 and ssp585 (Figure 8b,d). While the minimum suitability area was predicted for the period 2021–2040 ssp370, reaching an area of 0.35 × 10^7^ km^2^, with a decrease of 18.46% compared to that under near current climate conditions (Figure 8c). Overall, the trends for low suitable areas are similar in different future climate conditions.

**Figure 9:**
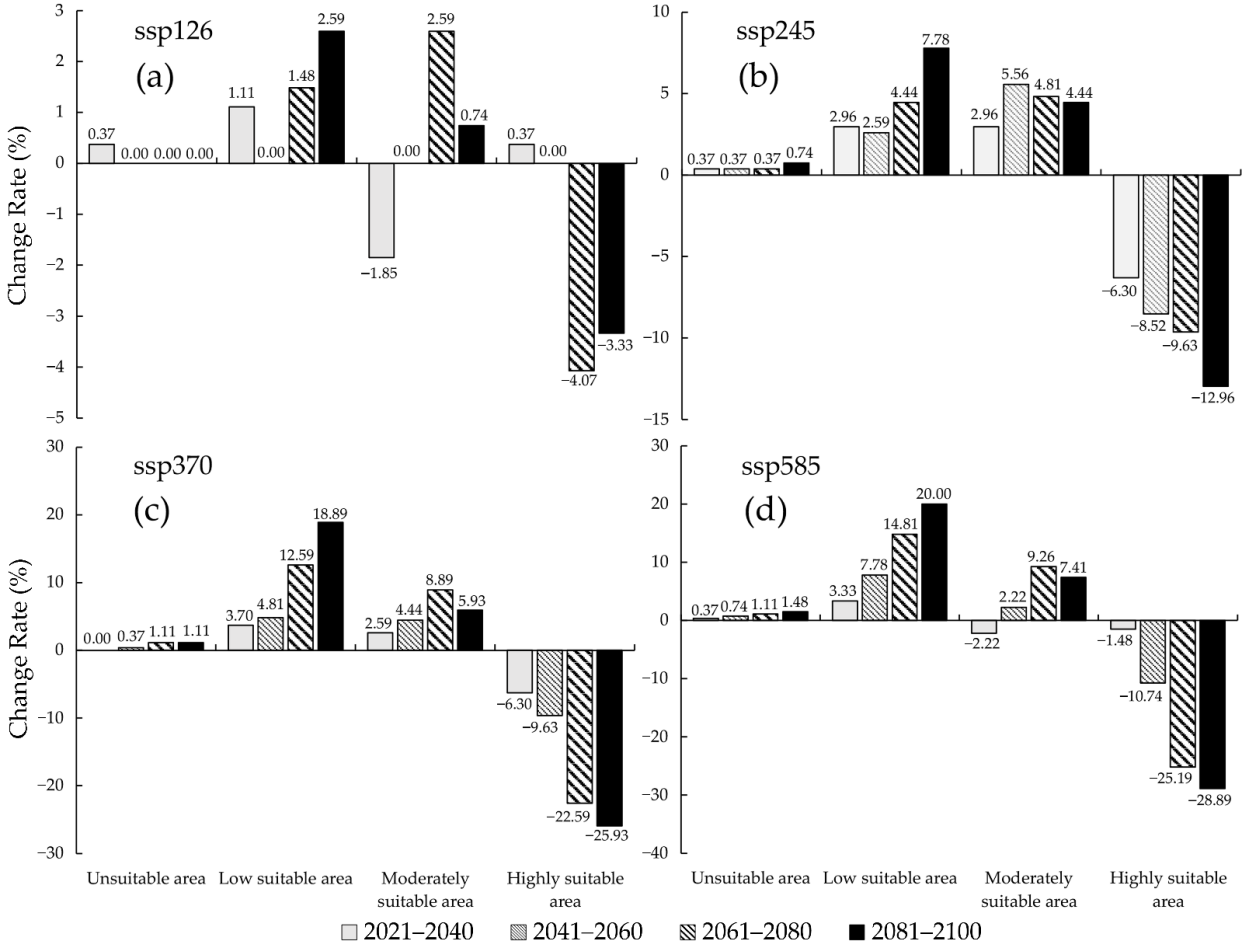
The change rate of predicted potential distribution of *S. juvencus* around the world under the future climate conditions during the periods of the 21st century under four CMIP6 climate scenarios, compared with the number of occurrence points under near current climate conditions and four shared socio-economic pathways: (**a**) ssp126; (**b**) ssp245; (**c**) ssp370; (**d**) ssp585.

Compared to the near current climatic condition, the actual global distribution, as presented by occurrence points, of *S. juvencus* under the future climatic condition, has changed (Figure 9). The number of occurrence points of *S. juvencus* is predicted to increase by 2.59% in moderately suitable areas under the future climatic condition 2061–2080, and in highly suitable areas it increases by 0.37% under the future climatic condition 2081–2100 (Figure 9a). Curiously, the actual number of occurrence points in highly suitable areas shows a decrease under the various future climate scenarios (Figure 9b–d). For the moderately suitable areas, there is a general increase in the number of occurrence points under the future climatic conditions, with the highest change recorded during the periods 2061–2080 ssp370 (Figure 9c) and 2061–2080 ssp585 (Figure 9d).

## 4. Discussion

In this study, we found that the wood wasp, *S. juvencus*, possesses highly suitable areas in the Northern Hemisphere and can spread to previously unsuitable and moderately suitable areas under future climate scenarios in the 21st century. As an invasive alien species, *S. juvencus* has been introduced intentionally or unintentionally by humans to regions outside their autochthonous geographic distribution, including New Zealand and parts of North America [27,55,56,57]. Like all wood wasps, the species is easily moved in solid wood packing materials with or without bark used in trade; its larvae and pupae can be introduced and co-infest host plants along with other wood wasps. Given the species’ adaptability to temperature and precipitation, suitable areas may experience severe infestation, leading to high economic losses, especially in the timber industry.

From 2016 to 2020, we have revisited some of the reported records of Chinese and European species, deleting and correcting old records and adding new ones. We further found that *S. juvencus* could be easily caught in traps for bark beetles and long-horned beetles, which seemed to be a common pattern of wood wasps associated with other wood borers.

### 4.1. The Association between Environmental Variables and the Potential Spread of S. juvencus

We found that the worldwide distribution of *S. juvencus* is mainly impacted by monthly average maximum temperature in February (tmax2), which influenced the species’ distribution and contributed unique information in predicting area suitability. The following were the maximum temperatures of the warmest month (bio5), monthly average minimum temperature in July (tmin7), and monthly total precipitation in June (prec6), suggesting strong environmental adaptability driven by temperature (tmax2, tmin7, bio5, and bio7) and precipitation (prec6, prec9, and bio14) (Figure 5).

In this study, the suitable distribution areas and unsuitable distribution areas were defined by the lowest presence threshold (LPT). The range of predicted suitable habitats for *S. juvencus* under near current climatic conditions was 80° N–60° S (Figure 3b). The predicted suitable habitats for *S. noctilio* were 30°–60° N and 25°–55° S [30]; thus, when compared to *S. noctilio*, *S. juvencus* seems to have a larger area of suitable habitats, especially in Europe. However, *S. noctilio*, as an invasive species native to Eurasia, has a wider spread around the world, while *S. juvencus* has widely been established in the Northern Hemisphere. It is worth noting that *S. noctilio* and *S. juvencus* have many overlapping suitable habitats, potentially co-infesting the hosts and bringing them into the interspecific competition.

We found that the highly suitable area for *S. juvencus* under the near current climate covers a total area of 4.56 × 10^7^ km^2^, and is primarily driven by summer rainfall patterns, temperature, and winter temperature. These areas increase by a slight proportion when modeled in future climate scenarios. Changes in environmental factors, especially temperature and rainfall, can influence microclimates, such as air temperature, wind speed, relative humidity, solar radiation, and sky radiation. It further influences the habitability of areas for the species, which depends on species’ thermal tolerances, desiccation resistance, and metabolic processes [58,59]. The increased impact of climate change, particularly changes in rainfall and temperature patterns, can influence the species’ survival ability by impacting the reproductive output, larval survival, and survival of fungal obligate mutualism as in the case of *S. noctilio* [60]. In the lifecycle of *S. juvencus*, unfavorable temperatures can lead to poor survival of eggs and larvae. The eggs are usually overwintered, but some are hatched in late summer, and the larvae over winter in the first or second instar. The prepupal stage lasts 4 to 6 weeks and is followed by the pupal stage for 2 to 3 weeks. In North America, the emergence period of *S. juvencus* mainly occurs from late summer to late autumn [61], while in Europe, the peak emergence period of *S. juvencus* extends to summer [26]. In China, according to our investigation from 2015 to 2019, *S. juvencus* was collected on *Picea obovate*, *P. schrenkiana*, and *P. crassifolia* forests during the middle of July to early September. *S. juvencus* was found to co-infest the weakened or dying host plant species with other *Sirex* wood wasps, including *S. ermak*, *S. dux*, *Urocerus gigas*, and *Xeris spectrum* in China, which means the emergence periods of some wood wasps are overlapping. Being infested by several *Sirex* wood wasp species may accelerate tree death [62,63].

Temperature and precipitation have been shown to affect larvae, pupae, and the emergence of *S. juvencus* [64]. Thus, precipitation (prec6) and temperature (bio5 and tmin7) may affect the critical period that pupae emerge, and we show in this study that temperature is the most influential predictor of current and future distributions of *S. juvencus*. The biological characteristics of *S. noctilio* were used to estimate *S. juvencus* egg and larval development. The estimates indicated that immature larval stages developed progressively slower at temperatures below 15 °C and reached a lower threshold at about 6 °C under natural conditions. Complete development depends on temperatures occurring within the range of 12.5–33.5 °C [65], and very few *S. juvencus* emerged when the maximum daily temperature was lower than 20 °C. [61]. Besides, in China, according to our observation, *S. juvencus* mainly emerged from 12 am to 5 pm, which is the warmest period of the day. In the current study, the maximum monthly temperature in February (tmax2) and the maximum temperature in the warmest month (bio5) ranged between 3–20 °C and 3–36 °C, respectively; while the temperature (tmin7) during the emergence period of *S. juvencus* was 3–23 °C. In addition, *S. juvencus*, like other wood wasps, also forms an obligate mutualism association with a wood-rotting fungus, *A. areolatum*, which assists the larvae of *S. juvencus* in digesting plant cellulose, hemicellulose, or wood fibers. The larvae lack essential enzymes required for the digestion of these substances. The fungus, supported by the mucus, enables this by breaking down the cellulose and lignin in the wood, enabling the larvae to consume it [66]. The growth conditions of the obligate mutualistic fungus of *S. juvencus*, *A. areolatum*, are also affected by temperature. The growth condition of *A. areolatum* is limited to 10–30 °C, which overlaps with that of *Sirex* species [67]. Furthermore, the fungus prefers to grow in wood with a lower moisture content [68,69]. For another *Sirex* wood wasp, *S. nitobei*, the precipitation (prec7 and prec12) and temperature (tmax2 and tmin7), related to the periods of the emergence, growth of larvae and its obligate mutualistic fungus, also significantly impact its distribution [29]. This supports the result that temperature and precipitation suitability is critical in explaining the current and future distribution patterns of *S. juvencus*.

In comparison to the lifestyle of *S. noctilio*, *S. juvencus* is less cold-tolerant and requires much lower rainfall. For both species, larvae are highly cold-tolerant, with *S. noctilio* larvae able to supercool to −24.27 ± 0.62 °C [70], much lower than the suitable temperature of the maximum monthly temperature in February (tmax2) for *S. juvenctus* larvae (Figure 6a), thus possibly explaining some of the observed differences in distribution patterns.

By comparing with these results of suitable temperature for larvae and emergence obtained from the biological study and the maximum entropy model, it is evident that the physiological tolerance of *S. juvencus* to temperature is stronger than previously shown [71]. The rainfall also influenced the current and future distribution, *S. juvencus* prefers low rainfall areas and low moisture content in the host plant. Evidence indicates that the species also infested weak and dying trees that have already been infested by other wood wasps, which supports this finding that *S. juvencus* preferred lower moisture than other wood wasps. Furthermore, this could explain the spread of *S. juvencus* into moderately suitable areas as predicted by a maximum entropy model, and not always into highly suitable areas that may favor more dominant and competitive wood wasp species such as *S. noctilio*, which may also have higher thermal tolerances to extreme environments in its highly suitable areas.

### 4.2. The Main Factors Affecting the Predictive Accuracy of the Maximum Entropy Model

We found the maximum entropy model to be highly effective and accurate in predicting the potentially suitable areas of *S. juvencus*. The model yielded a high AUC value, and low ORmtp and OR10 (Table 3) [50,54]. Besides, only a slight deviation between the mean omission curve on the test data and the predicted omission (Figure 2) indicated that the predicted result’s accuracy is a high predictive ability. Using the AUC value to verify the maximum entropy model’s predictive accuracy is controversial. Even though the AUC value as an evaluation criterion of the maximum entropy model is widely used, it does present some problems: (1) it ignores the predicted probability values and the goodness-of-fit of the model; (2) it summarizes the test performance over regions of the ROC space in which one would rarely operate; (3) it weights omission and commission errors equally; (4) it does not give information about the spatial distribution of model errors, and, most importantly, (5) the total extent to which models are carried out highly influences the rate of well-predicted absences and the AUC value [51]. The AUC value could vary with the selected background points’ spatial range, with a more extensive spatial range resulting in a higher AUC value [52]. However, we did not find a significant difference between the AUC value and AUC ratios under different climate conditions in this study. The AUC values and AUC ratios were greater than 0.9 and 1.0, respectively, indicating that the predictive accuracy was a high predictive ability (Table 2). Moreover, it is important to incorporate up-to-date and relevant climate and environmental data when using distribution models. Here we used the updated WorldClim database with near current and future climate variables, which gave predictions that are much closer to reality and have biological relevance. However, there is still a 20-year gap with the current timeline, which means that there are still restrictions for predicting the potentially suitable area of the species as researchers cannot access the latest climate database.

### 4.3. The Worldwide Suitable Area of S. juvencus under the Future Climate Conditions and the Suggestions about the Pest Control

Comparing the range of the potentially suitable area of *S. juvencus* under the future climate conditions with that under the near current climate conditions, the area of the total suitable area was increasing under the four CMPI6 climate scenarios, with a slight increase of 0.37% in highly suitable areas under climate scenario 2021–2040 ssp126, an increase in land area from 9.03 × 10^7^ km^2^ to 9.23 × 10^7^ km^2^ of predicted potentially highly suitable areas. The percentage of increase in the area of the moderately suitable area was 9.26%, and that of the low suitable area was 20.00%, showing a more rapid increase in suitability under the scenarios 2061–2080 ssp585 and 2041–2060 ssp585; under the same climate scenarios, low suitable areas increased by 1.48%. Overall, under the climate scenarios 2081–2100 ssp585 (highly suitable area), 2021–2040 ssp126 (moderately suitable area), and 2041–2060 ssp126 (low suitable area), there is a rapid decrease in the highly suitable area and a general decrease in the moderately and low suitable areas, which could be associated with rainfall patterns and temperature variations in these periods and climate scenarios that would affect the lifecycle of *S. juvencus* as well as its obligate mutualistic fungus. The most apparent changes in the suitable areas were in Europe, North America, and Southwest China. In Europe, the suitable area moved towards the north. The area of the highly suitable area increased in northwestern North America and Southwest China. By comparing the change rate of existence probability of the current occurrence points, we can also analyze which CMIP6 climate scenario is more reasonable under different future climate conditions. In the climate scenarios 2081–2100 ssp370 (highly suitable area), 2021–2040 ssp245 (moderately suitable area), 2021–2040 ssp370 (low suitable area), and 2021–2400 ssp126 (unsuitable area), the increases were 37.79%, 28.14%, 3.30%, and 2.15%, respectively. Furthermore, in the climate scenarios 2021–2040 ssp370 (highly suitable area), 2061–2080 ssp585 (moderately suitable area), 2061–2080 ssp245 (low suitable area), and 2021–2400 ssp126 (unsuitable area), the decreases were 18.46%, 15.75%, 6.48%, and 1.13%, respectively. The time interval of the 21st century periods under four CMIP6 climate scenarios is ten years, and thus within ten years, the area of the suitable area would not change significantly. As a result, the potentially suitable area obtained under the scenario ssp126 might be a more realistic result.

The spread of *S. juvencus* could change with the climate change patterns associated with low rainfall and high temperatures. *S. juvencus* are highly adaptable and can spread quickly due to their distance dispersal abilities. *S. juvencus* showed high adaptability levels and are likely to have physiological tolerance to highly variable environmental conditions. The dependency of *S. juvencus* on its obligate mutualistic fungus can influence the potential survival in new areas. Since evidence suggests some dependencies on other *Sirex* species for host infestation, generalized pest control could also affect *S. juvencus*.

## 5. Conclusions

We found that the maximum entropy model is effective in predicting the potential global distribution of *S. juvencus* by predicting potential future climatic conditions relative to current distribution patterns. We have incorporated near current and future climate scenarios allowed for a better predictive outcome and enabled the precise identification of important environmental variables driving current and potential future suitable areas, which were mostly driven by temperature and precipitation. As expected, temperature and precipitation were very important in predicting the potentially suitable areas of *S. juvencus*, which could be related to the biological requirements for its whole life cycle and obligate mutualistic fungus. Under future climate conditions, the total area and the area of suitable areas for *S. juvencus* will increase as the species moves more into moderately suitable areas. The results indicate that the movement of the species into moderately suitable areas could be due to competition or dependency on co-infestation with other species or the limitation of rainfall. The area with moderate suitability could have much lower rainfall and could also be the site of abundant other *Sirex* wood wasp species that *S. juvencus* can co-infest the host plants with.

## Figures and Tables

**Table 1:** Environmental variables used in predicting the potential distribution of *S. juvencus*.

Climate Variables	Code	Unit
Annual mean temperature	bio1	°C
Mean diurnal range	bio2	°C
Isothermality	bio3	%
Temperature seasonality	bio4	—
Max temperature of warmest month	bio5	°C
Min temperature of coldest month	bio6	°C
Temperature annual range	bio7	°C
Mean temperature of wettest quarter	bio8	°C
Mean temperature of driest quarter	bio9	°C
Mean temperature of warmest quarter	bio10	°C
Mean temperature of coldest quarter	bio11	°C
Annual precipitation	bio12	mm
Precipitation of wettest month	bio13	mm
Precipitation of driest month	bio14	mm
Precipitation seasonality	bio15	—
Precipitation of wettest quarter	bio16	mm
Precipitation of driest quarter	bio17	mm
Precipitation of warmest quarter	bio18	mm
Precipitation of coldest quarter	bio19	mm
Monthly average minimum temperature	tmin1–tmin12	°C
Monthly average maximum temperature	tmax1–tmax12	°C
Monthly total precipitation	prec1–prec12	mm

**Table 2:** Environmental variables used in predicting the potential geographic distribution of *S. juvencus*.

Climate Variables	Unit	Percent Contribution
tmax2	°C	33.1
prec6	mm	12.9
bio5	°C	3.3
bio14	mm	3.1
tmin7	°C	2.7
prec9	mm	1.5
bio7	°C	1.5

**Table 3:** The predictive accuracy of the maximum entropy model estimated by AUC, ORmtp, OR10, and AUC ratio.

Climate Scenarios	AUC	ORmtp	OR10	AUC Ratio
Near current	0.954	0.005	0.119	1.80
2021–2040 ssp126	0.951	0.011	0.134	1.73
2041–2060 ssp126	0.959	0.002	0.124	1.72
2061–2080 ssp126	0.957	0.005	0.108	1.71
2081–2100 ssp126	0.955	0.010	0.140	1.73
2021–2040 ssp245	0.953	0.006	0.137	1.74
2041–2060 ssp245	0.956	0.005	0.119	1.72
2061–2080 ssp245	0.954	0.007	0.133	1.70
2081–2100 ssp245	0.957	0.003	0.101	1.69
2021–2040 ssp370	0.958	0.003	0.093	1.74
2041–2060 ssp370	0.954	0.005	0.116	1.71
2061–2080 ssp370	0.956	0.005	0.117	1.64
2081–2100 ssp370	0.955	0.008	0.127	1.56
2021–2040 ssp585	0.952	0.009	0.127	1.72
2041–2060 ssp585	0.956	0.005	0.131	1.67
2061–2080 ssp585	0.953	0.008	0.142	1.57
2081–2100 ssp585	0.955	0.004	0.123	1.54
